# Dental education experiences, career outlook, and professional preparedness: a cross-sectional study of senior students at private universities in Iraq

**DOI:** 10.3389/fdmed.2025.1713400

**Published:** 2026-01-06

**Authors:** Fouad Y. H. Al-Sudani, Ausama A. Fathallh, Mohammad I. Sheiaa, Shaymaa K. Hassoon, Sabreen S. Abed Almuhssen, Raghad I. Kadhum, Hiba kh. Abdullah

**Affiliations:** 1 College of Dentistry, Al-Esraa University, Baghdad, Iraq; 2 College of Dentistry, Ashur University, Baghdad, Iraq

**Keywords:** dental education, dental practice, private university, preparedness, career prospects

## Abstract

**Background:**

Senior dental students’ transition from preclinical education to clinical practice is critical for their preparedness and future career outlook. In Iraq, private dental institutions are increasingly contributing to the dental workforce, but little is known about how well they prepare students for professional practice. This study aims to assess senior dental students’ perceptions of professional preparedness and career outlook at selected private universities in Iraq, and to identify the educational and demographic factors associated with these perceptions.

**Materials and methods:**

A cross-sectional survey was conducted in February 2025 among fourth- and fifth-year dental students at three private universities in Baghdad (Al-Esraa, Ashur, and Uruk). A purposive sampling technique was used. The sample size was 1,180 eligible students, of whom 1,041 responded (response rate: 88%). A bilingual, paper-based questionnaire was distributed, covering demographics, educational experiences, and career expectations. Data were analyzed using descriptive statistics, multinomial, and ordered logistic regression models, adjusting for demographic and socioeconomic factors.

**Results:**

A total of 1,041 senior dental students (38.3% males, 61.7% females) participated, mostly aged 21–23 years (85.1%) and single (92.9%). Over half (55.8%) viewed the dental profession as declining, while 48.9% felt somewhat prepared for practice. Significant gender differences appeared in marital and employment status (*p* < 0.05). Prioritizing job stability (RRR = 2.5, *p* < 0.001), salary (RRR = 3.0, *p* < 0.001), and work–life balance (RRR = 2.5, *p* < 0.001) predicted pessimism. Students rating education as excellent (RRR = 0.1, *p* < 0.001) or good (RRR = 0.3, *p* = 0.031) were less likely to perceive decline and more likely to feel prepared (OR = 3.9, *p* < 0.001). Positive views of private education, experienced faculty (OR = 1.6, *p* = 0.001), and job stability (OR = 1.3, *p* = 0.035) further enhanced preparedness and optimism.

**Conclusion:**

Students’ perceptions of educational quality and career values significantly influenced their professional outlook and preparedness for practice, highlighting the role of educational experience and career priorities in shaping attitudes toward the dental profession. Incorporating mentorship and market-readiness modules into the curriculum may bridge the gap between clinical competence and employability among future dental graduates.

## Introduction

The final years of dental education mark a pivotal phase in the professional growth of dental students. During this period, they transition from preclinical settings to hands-on patient care, starting to define their career directions in clinical practice, specialization, academia, or public health. This important transition is shaped by individual goals and the educational experiences accumulated throughout their training. From this perspective, it is essential to understand how educational elements influence students’ career perspectives and their sense of preparedness for professional practice. This understanding is key to enhancing dental curricula and ensuring graduates are well equipped to deal with the challenges of contemporary dentistry.

Professional preparedness refers to the extent to which dental graduates acquire the knowledge, technical skills, and professional attitudes required to begin independent clinical practice safely and competently. It encompasses not only clinical and technical competence but also communication skills, ethical reasoning, decision-making, and professional confidence developed through undergraduate training (1–3). Preparedness is often measured through self-reported perceptions of readiness for clinical practice, as well as structured competency evaluations and faculty assessments (1, 4). Ensuring high professional preparedness is vital for maintaining patient safety, promoting career satisfaction, and supporting the transition from student to practicing clinician (1, 5). Conversely, inadequate preparedness may compromise patient outcomes, hinder clinical performance, and reduce graduates’ confidence when entering the dental workforce (4, 6).

Career outlook reflects dental students’ expectations and perceptions regarding their professional future, including employment opportunities, job security, financial rewards, and the social prestige of dentistry (7–9). It also includes preferences for specific career paths, such as general practice, specialization, or academic and public health roles (7, 10). A positive career outlook promotes motivation, persistence, and long-term engagement in the profession (9, 11), whereas pessimistic views about the profession's future—such as concerns over unemployment or declining income—can lead to reduced morale and professional dissatisfaction (12, 13). Understanding dental students’ career outlook helps educators and policymakers identify potential workforce challenges, align curricula with job market realities, and sustain the attractiveness of the profession (8, 14).

In Iraq, dental education has undergone major expansion in the past two decades. Before 2003, dental training was offered exclusively in public universities under standardized governmental supervision (15). Following political and economic reforms, the Ministry of Higher Education and Scientific Research authorized the establishment of private universities to meet growing demand for medical and dental education. Currently, more than sixty private dental schools operate across the country (15, 16). While these institutions have increased access to dental education, they face challenges related to faculty experience, clinical case availability, curriculum standardization, and regulatory oversight (16, 17). Rapid growth in the number of graduates has also contributed to concerns over job market saturation and employment instability among young dentists (13, 16). As private institutions now account for a substantial proportion of Iraq's dental workforce, assessing their educational effectiveness and outcomes has become a national priority (15).

Despite the significant role of private universities in Iraq's dental education sector, there is limited research examining how effectively they prepare students for professional dental practice and how their educational experiences influence career outlook. Previous studies in other regions have highlighted that students’ perceptions of educational quality, faculty support, and clinical exposure strongly affect their confidence, preparedness, and optimism toward their careers (1, 2, 5, 18, 19). However, similar data from Iraq's private universities remain scarce. Given the increasing number of dental graduates and the perceived decline in employment opportunities, it is important to evaluate how students perceive their readiness for practice and the future of their profession. Such evidence can guide academic reforms, inform accreditation policies, and support better alignment between dental education and labour market needs in Iraq (15, 16, 20).

The present study aims to assess senior dental students’ perceptions of professional preparedness and career outlook at selected private universities in Iraq, and to identify the educational and demographic factors associated with these perceptions.

## Materials and methods

### Study design and population

The current study employed a cross-sectional design to assess senior dental students’ perceptions of their professional preparedness and career outlook. The target population included all fourth- and fifth-year undergraduate dental students enrolled at three private universities in Baghdad, Iraq: Al-Esraa University (https://www.esraa.edu.iq/), Ashur University (https://au.edu.iq/), and Uruk University (https://www.uruk.edu.iq/). These universities were selected because they represent well-established private institutions with large dental programs and diverse student populations.

### Time and place of the study

Data collection was conducted during February 2025 in Baghdad, Iraq. Official permissions to carry out the research were obtained from the participating Colleges of Dentistry at Al-Esraa University, Ashur University, and Uruk University in November 2024.

### Sampling technique

A purposive sampling technique was used to include all available senior students (fourth- and fifth-year) who were present during data collection sessions. This approach was selected to ensure a comprehensive representation of students who had completed most of their preclinical and clinical training and were preparing for entry into professional practice.

### Sample size

A total of 1,180 students were eligible to participate in the study across the three universities. Out of these, 1,041 students completed the questionnaire, with a response rate of 88%. This high participation rate ensured adequate statistical power and representativeness of the study population.

### Sampling procedures

Data collection was conducted in person using a paper-based, self-administered questionnaire. Students were approached during their scheduled lectures and clinical sessions after obtaining permission from course instructors. Each student received a printed questionnaire along with an explanation of the study objectives and assurances of anonymity and voluntary participation. Completed questionnaires were collected on the same day to ensure a high response rate and minimize nonresponse bias.

### Data collection tool

Data were collected using a structured, bilingual (English and Arabic) questionnaire specifically developed for this study. The questionnaire was adapted from previously validated surveys that explored dental education quality, student preparedness, and professional expectations in similar contexts (8, 20, 21). It included both closed-ended and multiple-choice questions designed to capture demographic, educational, and perceptual variables.

### Validity and reliability

The study's validity was ensured through several methodological and analytical procedures. The questionnaire was developed based on previously published surveys and adapted to the Iraqi dental education context, supporting its content validity. A bilingual format (English and Arabic) and pilot testing on a sample of students ensured clarity and cultural relevance, enhancing face validity. Internal validity was strengthened by the high response rate (88%) and the use of multinomial and ordered logistic regression models adjusting for key demographic and socioeconomic confounders. External validity was supported by the large sample size across three private universities, although generalizability is limited to similar educational settings in Iraq. Reliability was promoted through standardized administration and clear item wording; however, internal consistency coefficients (e.g., Cronbach's alpha) were not calculated, as most constructs were assessed using single-item measures. Overall, the instrument demonstrated acceptable validity and reliability for a cross-sectional educational study.

The questionnaire's construct validity was established through adaptation from previously validated studies (8, 20, 21), expert content review and pilot testing to confirm that items appropriately reflected the constructs of educational quality, preparedness and carer expectations.

### Questionnaire language

The questionnaire was developed in English and then translated into Arabic using a forward–backward translation method. Two bilingual experts translated the instrument into Arabic, and another independent translator performed the back-translation into English. The original and back-translated versions were compared to ensure conceptual equivalence and linguistic accuracy. This bilingual approach allowed students to respond in their preferred language, reducing misinterpretation and ensuring inclusivity. The questionnaire was pre-assessed for clarity and response choices on a group of students (ten students). It is important to mention that the teaching language in the three dental colleges is English throughout the five years. Therefore, students are already well accustomed to read and understand English texts and questions. The students received an explanation that participating in this research is not compulsory and is done anonymously. The participation in the study was voluntary, and the students had the right to refuse to take part in the study with no consequences whatsoever for them.

### Questions and variables

The questionnaire consisted of four main sections. The first section included eleven questions regarding demographic information (stage, age, gender, marital status, currently working, father's job, education level, income level, mother's job, education level, income level, and main source of study fee). The second section is composed of five questions concerning educational information, including questions about rating education, strength of dental education program, preparedness for a successful career in dentistry, comparison of private and public schools in terms of quality of education and career prospects, and students’ preferred specialty.

The third section included nine questions related to career expectations, intentions and job market conditions, future practice plan, possibility of getting hired in public job, expectation of unemployment period after graduation, best sector to offer employment opportunities for dental graduates, factor influencing choosing future career, challenges to secure employment after graduation, interest in pursuing postgraduate studies, structure of postgraduate studies, factors that influence postgraduate speciality choice.

The fourth section included seven questions in regard to information of post-graduation support, professional development. the questions included career counselling, practical experience, professional development opportunities, preparedness for the transition to professional practice, plans of retirement, future of the dental profession in Iraq, and improvement of the Iraqi dental healthcare system. The overall response rate of three universities was 88% (total number of students 1,180), as only 1,041 responded to the study invitations and filled out the questionnaire. It is important to note that the current research explored only information in second and fourth sections as the rest of the questionnaire items are beyond the current study's focus. Information about career outlook was gathered from the question: “What do you think is the future of the dental profession in Iraq? With answers as follow: Positive and growing, stable with slow growth, declining, not sure”. The data regarding professional preparedness were taken from the question: “How prepared do you feel for the transition from dental school to professional practice? With answers as follow: very prepared, somewhat prepared, not prepared, not sure”.

Responses from related items were grouped into thematic domains corresponding to educational experiences, career expectations, and program strengths. Categorical variables (e.g., gender, marital status) were coded numerically for analysis. Perception-related items were grouped and treated as ordinal variables where appropriate (e.g., rating of education quality). This organization facilitated regression modelling and allowed identification of associations between educational predictors and outcome variables.

### Data analysis

Data were entered and cleaned using Microsoft Excel^TM^ (Microsoft Corp., Redmont,WA, USA) and analyzed with Stata Statistical Software, Release 14.2 (StataCorp, College Station, TX, USA). Descriptive statistics were presented as frequencies and percentages for categorical variables. Chi-square tests were used to examine bivariate associations. To identify independent predictors, multinomial logistic regression was employed for categorical outcomes, and ordered logistic regression was used for ordinal outcomes. Results were expressed as Relative Risk Ratios (RRR) and Odds Ratios (OR) with 95% confidence intervals (CI) and *P*-values. All models were adjusted for potential confounders, including demographic and socioeconomic characteristics. All descriptive, bivariate, and multivariate analyses were conducted using the same version (Stata 14.2) to ensure consistency of statistical procedures and reproducibility.

### Ethical issues

Ethical approval for this study was obtained from the Ethical Committee of Al-Esraa University and further endorsed by the Higher Committee of Ethics at the Iraqi Ministry of Higher Education and Scientific Research. Informed consent was obtained from all participants before data collection. Participation was entirely voluntary, and students could withdraw at any time without penalty. Anonymity and confidentiality of responses were strictly maintained throughout the research process.

### Data availability statement

The data underlying this article will be shared on request to the corresponding author.

## Results

A total of 1,041 senior dental students participated in the study, comprising 399 males (38.3%) and 642 females (61.7%). The participants’ ages ranged from 21 to 30 years, with 886 students (85.1%) between 21 and 23 years, 139 students (13.4%) between 24 and 26 years, and 16 students (1.5%) between 27 and 30 years. Regarding university distribution, 673 students (64.7%) were from Al-Esraa University, 198 (19.0%) from Uruk University, and 170 (16.3%) from Ashur University. Students were almost evenly distributed across academic stages, with 576 (55.3%) in the fourth year and 465 (44.7%) in the fifth year.

In terms of marital status, 967 students (92.9%) were single, 68 (6.5%) were married, and 6 (0.6%) were divorced. Employment status showed that 849 students (81.6%) were not working, while 192 (18.4%) were working during their studies. Concerning tuition fee sources, 796 students (76.5%) relied on parental or personal funds, 197 (18.9%) on scholarships, and 48 (4.6%) on loans or other sources. For fathers’ educational levels, 163 (15.7%) had less than a high school education, 256 (24.6%) had completed high school, 508 (48.8%) had a university degree, 60 (5.8%) held a master's degree, and 54 (5.2%) held a PhD. For mothers, 283 (27.2%) had less than a high school education, 295 (28.3%) completed high school, 392 (37.7%) had a university degree, 45 (4.3%) held a master's degree, and 26 (2.5%) held a PhD.

Regarding perceptions of the dental profession outlook, 581 students (55.8%) viewed it as declining, 302 (29.0%) as stable with slow growth, 97 (9.3%) as positive and growing, and 61 (5.9%) were unsure. In terms of preparedness for professional dental practice, 336 students (32.3%) felt very prepared, 509 (48.9%) felt somewhat prepared, 131 (12.6%) felt not prepared, and 65 (6.2%) were unsure about their readiness. Statistically significant gender differences were observed in marital status, employment status, and father's level of education. Female students were more likely to be married and less likely to be employed, whereas male students were more commonly employed and had fathers with higher levels of educational attainment (Table 1).

**Table 1:** Demographic, socioeconomic, educational characteristics of dental students of three universities.

Characteristic	Categories	Male	Female	Total *N* (%)
Gender		399 (38.3%)	642 (61.7%)	1,041 (100%)
Age	21–23 years old	332 (37.5%)	554 (62.5%)	886 (85.1%)
	24–26 years old	61 (43.9%)	78 (56.1%)	139 (13.4%)
	27–30 years old	6 (37.5%)	10 (62.5%)	16 (1.5%)
University	Al-Esraa University	261 (38.8%)	412 (61.2)	673 (64.7%)
	Uruk University	69 (34.8%)	129 (65.2%)	198 (19.0%)
	Ashur University	69 (41.6%)	101 (59.4%)	170 (16.3%)
Student's stage	4th stage	230 (39.9%)	346 (60.1%)	576 (55.3%)
	5th stage	169 (36.3%)	296 (63.7%)	465 (44.7%)
Marital Status*	Single	380 (39.3%)	587 (60.7%)	967 (92.9%)
	Married	14 (20.6%)	54 (79.4%)	68 (6.5%)
	Divorced	5 (83.3%)	1 (16.7%)	6 (0.6%)
Currently working*	No	270 (31.8%)	579 (68.2%)	849 (81.6%)
	Yes	129 (67.2%)	63 (32.8%)	192 (18.4%)
Tuition fee source	Parental or own money	304 (76.2%)	492 (76.6%)	796 (76.5%)
	Scholarship	70 (17.5%)	127 (19.8%)	197 (18.9%)
	Loan or others	25 (6.3%)	23 (3.6%)	48 (4.6%)
Father's education*	Less than high school	54 (13.5%)	109 (17.0%)	163 (15.7%)
	High school	83 (20.8%)	173 (27.0%)	256 (24.6%)
	University	200 (50.1%)	308 (48.0%)	508 (48.8%)
	Masters	31 (7.8%)	29 (4.5%)	60 (5.8%)
	PhD	31 (7.8%)	23 (3.6%)	54 (5.2%)
Mother's education	Less than high school	94 (23.6%)	189 (29.4%)	283 (27.2%)
	High school	111 (27.8%)	184 (28.7%)	295 (28.3%)
	University	158 (39.6%)	234 (36.4%)	392 (37.7%)
	Masters	22 (5.5%)	23 (3.6%)	45 (4.3%)
	PhD	14 (3.5%)	12 (1.9%)	26 (2.5%)
Self-perceived dental profession outlook	Positive & growing	40 (10.0%)	57 (8.9%)	97 (9.3%)
	Stable with slow growth	102 (25.6%)	200 (31.2%)	302 (29.0%)
	Declining	233 (58.4%)	348 (54.1%)	581 (55.8%)
	Not sure	24 (6.0%)	37 (5.8%)	61 (5.9%)
Preparedness for professional dental practice	Very prepared	135 (33.8%)	201 (31.3%)	336 (32.3%)
	Somewhat prepared	188 (47.1%)	321 (50%)	509 (48.9%)
	Not prepared	48 (12.0%)	83 (12.9%)	131 (12.6%)
	Not sure	28 (7.0%)	37 (5.8%)	65 (6.2%)

* Chi square test, *P* < 0.05.

Based on the multinomial logistic regression analysis, several factors significantly influenced the perceived outlook of the dental profession. The adjusted models showed that students prioritizing job stability/security were 2.2 times more likely to view the dental profession as “Stable with slow growth” (Adjusted RRR = 2.2, 95% CI: 1.3–3.7, *P* = 0.002) and 2.5 times more likely to view it as “Declining” (Adjusted RRR = 2.5, 95% CI: 1.5–4.1, *P* = 0.000) compared to the reference group (positive and growing). Likewise, Students valuing salary/compensation were 1.6 times more likely to hold the view “stable with slow growth” (Adjusted RRR = 1.6, 95% CI: 1.1–2.3, *P* = 0.019) and 3.0 times more likely to hold the view “Declining” (Adjusted RRR = 3.0, 95% CI: 2.1–4.2, *P* = 0.000). Moreover, work-life balance was also associated with a 1.5 times higher likelihood of perceiving “Stable with slow growth” (Adjusted RRR = 1.5, 95% CI: 1.0–2.2, *P* = 0.037) and a 2.5 times higher likelihood of perceiving a “Declining” outlook (Adjusted RRR = 2.5, 95% CI:1.8–3.7,*P* = 0.000). Participants prioritizing location were 1.7 times more likely (Adjusted RRR = 1.7, 95% CI: 1.1–2.7, *P* = 0.013), and those concerned with opportunities for professional development were 1.6 times more likely (Adjusted RRR = 1.6, 95% CI: 1.0–2.4, *P* = 0.048), to perceive the profession's outlook as “Declining”. The reputation of the institution/clinic did not show a statistically significant association with the dental profession outlook in the adjusted model (Table 2).

**Table 2:** Multinomial logistic regression models of the association of factors of future career and rating of how well the curriculum prepared them for a successful career and dental profession outlook.

Important factors when considering future career	Self-perceived dental profession outlook^a^					
	Stable with slow growth		Declining		Not sure	
	Unadjusted RRR (95%CI)	Adjusted RRR^b^ (95%CI)	Unadjusted RRR (95%CI)	Adjusted RRR^b^ (95%CI)	Unadjusted RRR (95%CI)	Adjusted RRR^b^ (95%CI)
Job stability/security	**4.0 (2.5–6.4)**	**2.2 (1.3–3.7)**	**7.3 (4.7–11.4)**	**2.5 (1.5–4.1)**	**0.8 (0.4–1.5)**	1.2 (0.6–2.2)
*P*-value	**0.000**	**0.002**	**0.000**	**0.000**	**0.528**	0.652
Location	**2.6 (1.7–4.0)**	1.3 (0.8–2.0)	**5.5 (3.7–8.2)**	**1.7 (1.1–2.7)**	**0.5 (0.3–0.9)**	0.7 (0.3–1.4)
*P*-value	**0.000**	0.261	**0.000**	**0.013**	**0.038**	0.304
Opportunities for Professional development	**2.8 (1.8–4.2)**	1.4 (0.9–2.2)	**5.1 (3.5–7.6)**	**1.6 (1.0–2.4)**	0.6 (0.3–1.0)	0.8 (0.4–1.4)
*P*-value	**0.000**	0.158	**0.000**	**0.048**	0.061	0.400
Reputation of institution/clinic	**2.6 (1.7–4.0)**	1.3 (0.8–2.1)	**4.9 (3.3–7.3)**	1.4 (0.9–2.3)	**0.4 (0.2–0.9)**	0.6 (0.3–1.2)
*P*-value	**0.000**	0.275	**0.000**	0.090	**0.016**	0.140
Salary/compensation	**2.9 (2.1–4.0)**	**1.6 (1.1–2.3)**	**6.7 (5.1–8.9)**	**3.0 (2.1–4.2)**	**0.5 (0.4–0.9)**	0.7 (0.4–1.2)
*P*-value	**0.000**	**0.019**	**0.000**	**0.000**	0.012	0.282
Work-life balance	**2.9 (2.1–4.1)**	**1.5 (1.0–2.2)**	**6.7 (4.9–9.1)**	**2.5 (1.8–3.7)**	0.6 (0.4–1.0)	0.8 (0.5–1.4)
*P*-value	**0.000**	**0.037**	**0.000**	**0.000**	0.052	0.510
Rating of how well the dental program prepared student for a successful career)^#^						
Yes, very well	0.9 (0.2–4.8)	0.9 (0.2–5.0)	0.4 (0.1–1.7)	0.4 (0.1–1.8)	**0.1 (0.02–0.6)**	**0.1 (0.02–0.6)**
*P*-value	0.937	0.947	0.206	0.231	**0.014**	**0.014**
Yes, somewhat	1.2 (0.2–6.2)	1.2 (0.2–6.0)	0.6 (0.1–2.8)	0.6 (0.1–2.9)	**0.2 (0.03–0.9)**	**0.1 (0.03–0.8)**
*P*-value	0.812	0.849	0.562	0.558	**0.042**	**0.028**
No, not enough	0.8 (0.2–4.5)	0.8 (0.1–4.3)	1.1 (0.2–4.9)	1.1 (0.2–4.9)	0.2 (0.03–1.0)	**0.2 (0.03–0.9)**
*P*-value	0.854	0.800	0.933	0.935	0.053	**0.037**

Bold values are statistically significant.

a Reference group (positive & growing), RRR, relative-risk ratios; CI, confidence interval.

b Adjusted for demographics (Age, gender, marital status) and socioeconomic status (currently working (yes, no), study money source (own or parental money, scholarship, loan or others).

# Reference group (Not sure).

Dental students who felt very well prepared (Adjusted RRR = 0.1, 95% CI: 0.02–0.6, *P* = 0.014) and somewhat prepared (Adjusted RRR = 0.1, 95% CI: 0.03–0.8, *P* = 0.028) and not enough prepared (Adjusted RRR = 0.2, 95% CI: 0.03–0.9, *P* = 0.037) were significantly less likely to be “Not sure” about the dental profession outlook compared to the reference group. There were no statistically significant associations between feeling not enough prepared and the perceived dental profession outlook in the Adjusted models for “Stable with slow growth” or “Declining,” Moreover, demographic and socioeconomic factors played a significant role (changing the parameters from statistically significant into non-significant) particularly in the association of ‘Important factors when considering future career’ (location, opportunities for professional development, and reputation of the institution/clinic) and Self-perceived dental profession outlook (Table 2).

Students who rated their dental education as “Excellent” (Adjusted RRR = 0.1, 95%CI:0.03–0.3, *p* = 0.000) or “Good” (Adjusted RRR = 0.3, 95%CI:0.08–0.9, *p* = 0.031) were significantly less likely to perceive the dental profession outlook as “Declining.” Regarding Perceived Educational & Career Advantage, those who believed that private dental schools offer better opportunities than public ones, in terms of quality of education and career prospects, also showed a significantly decreased relative risk of a “Declining” outlook (Adjusted RRR = 0.5, 95%CI:0.2–0.8, *p* = 0.015) compared to the reference group (Table 3).

**Table 3:** Multinomial logistic regression models of the association of several educational factors and self-perceived dental profession outlook.

Rate the quality of dental education (Independent)	Self-perceived dental profession outlook^a^					
	Stable with slow growth		Declining		Not sure	
	Unadjusted RRR (95%CI)	Adjusted RRR^b^ (95%CI)	Unadjusted RRR (95%CI)	Adjusted RRR^b^ (95%CI)	Unadjusted RRR (95%CI)	Adjusted RRR^b^ (95%CI)
Excellent	1.4 (0.3–6.5)	1.6 (0.3–7.7)	**0.09 (0.02–0.3)**	**0.1 (0.03–0.3)**	**0.2 (0.04–0.9)**	0.2 (0.04–1.1)
*P*-value	0.645	0.513	**0.000**	**0.000**	**0.043**	0.070
Good	1.7 (0.4–7.2)	1.7 (0.4–7.3)	**0.3 (0.08–0.9)**	**0.3 (0.08–0.9)**	**0.2 (0.05–0.9)**	**0.2 (0.05–0.9)**
*P*-value	0.490	0.484	**0.029**	**0.031**	**0.033**	**0.034**
Average	3.3 (0.7–15.2)	3.2 (0.7–14.7)	0.6 (0.2–2.2)	0.6 (0.2–2.2)	0.7 (0.2–3.4)	0.7 (0.1–3.2)
*P*-value	0.125	0.138	0.476	0.492	0.711	0.648
Perceptions of Private vs. Public Dental Schools: Education quality& Career Prospects						
Worse	1.1 (0.4–2.5)	1.1 (0.5–2.6)	1.7 (0.8–3.9)	1.8 (0.8–4.1)	1.2 (0.4–3.4)	1.3 (0.4–3.7)
*P*-value	0.875	0.819	0.178	0.142	0.734	0.650
About the same	1.3 (0.7–2.5)	1.4 (0.7–2.6)	1.0 (0.5–1.9)	1.1 (0.6–2.0)	0.8 (0.4–2.0)	0.9 (0.4–2.0)
*P*-value	0.379	0.342	0.942	0.813	0.734	0.786
Better	0.6 (0.3–1.1)	0.6 (0.3–1.2)	**0.5 (0.2–0.8)**	**0.5 (0.2–0.9)**	**0.1 (0.03–0.6)**	**0.1 (0.03–0.8)**
*P*-value	0.117	0.146	**0.014**	**0.015**	**0.000**	**0.000**
Strengths of dental education program						
Experienced faculty	0.8 (0.5–1.4)	1.0 (0.6–1.7)	0.7 (0.4–1.1)	0.8 (0.5–1.3)	**0.2 (0.08–0.6)**	**0.3 (0.09–0.7)**
*P*-value	0.556	0.967	0.151	0.415	**0.003**	**0.009**
Modern facilities &equipment	0.6 (0.3–1.2)	0.7 (0.4–1.3	0.7 (0.4–1.3)	0.8 (0.4–1.4)	0.6 (0.2–1.5)	0.7 (0.3–1.7)
*P*-value	0.152	0.271	0.310	0.437	0.296	0.417
Practical/Clinical training	0.9 (0.5–1.6)	0.9 (0.5–1.6)	1.0 (0.6–1.6)	1.0 (0.6–1.6)	1.0 (0.5–2.2)	1.0 (0.4–2.2)
*P*-value	0.858	0.838	0.903	0.933	0.960	0.998
Research opportunities	0.5 (0.2–1.1)	0.5 (0.2–1.2)	0.5 (0.3–1.1)	0.6 (0.3–1.1)	0.5 (0.1–1.6)	0.5 (0.1–1.7)
*P*-value	0.096	0.121	0.087	0.120	0.246	0.290
Strong theoretical foundation	0.7 (0.4–1.2)	0.7 (0.4–1.2)	0.9 (0.6–1.4)	0.9 (0.6–1.5)	0.9 (0.4–1.8)	0.9 (0.4–1.8)
*P*-value	0.180	0.262	0.679	0.794	0.719	0.758

Bold values are statistically significant.

a Reference group (Positive & growing), RRR, relative-risk ratios; CI, confidence interval.

b Adjusted for demographics (Age, gender, marital status) and socioeconomic status (currently working (yes, no), study money source (own or parental money, scholarship, loan or others).

Regarding dental profession outlook being “Not sure”, multiple factors were associated with a reduced relative risk. Rating dental education quality as “Excellent” (Adjusted RRR = 0.2, 95% CI; 0.04–0.9, *p* = 0.043) or “Good” (Adjusted RRR = 0.2, 95% CI: 0.05–0.9, *p* = 0.033) was linked to a lower likelihood of being “Not sure.” The belief that private dental schools offer better opportunities than public ones was also strongly associated with a reduced relative risk of a “Not sure” outlook (Adjusted RRR = 0.1,95% CI: 0.03–0.6, *p* = 0.000). Finally, perceiving ‘Experienced faculty’ as a strength of their dental education program was associated with a significantly lower relative risk of individuals reporting a “Not sure” profession outlook (Adjusted RRR = 0.3, 95% CI:0.09–0.8, *p* = 0.009) (Table 3).

Dental students who rated dental education quality as “excellent” had significantly higher odds of feeling very prepared compared to those who rated it as poor (Adjusted OR = 3.9, 95% CI: 2.2–7.0, *P* < 0.001). Those who rated it as good (Adjusted OR = 2.6, 95% CI: 1.6–4.3, *P* < 0.001) or average (Adjusted OR = 1.9, 95% CI: 1.1–3.1, *P* = 0.013) also reported higher preparedness for dental practice. Students who believed that the dental program prepared them “very well” for a successful career had significantly higher odds of feeling ready for practice (Adjusted OR = 3.3, 95% CI: 1.5–7.0, *P* = 0.002). Other associations were not statistically significant The students who felt their private school gave them a better opportunities than public ones in terms of education quality and career prospects, had over twice odds of feeling well-prepared (Adjusted OR = 2.1,95% CI: 1.5–3.0, *P* = 0.000. other groups showed no significant difference in preparedness (Table 4).

**Table 4:** Ordered logistic regression models of educational factors and preparedness for professional practice.

Rate the quality of dental education	Preparedness for professional dental practice	
	Unadjusted OR (95% CI)	Adjusted OR (95% CI)^a^
Excellent	**4.2 (2.3–7.4)**	**3.9 (2.2–7.0)**
*P*-value	**0.000**	**0.000**
Good	**2.6 (1.6–4.2)**	**2.6 (1.6–4.3)**
*P*-value	**0.000**	**0.000**
Average	**1.8 (1.1–3.0)**	**1.9 (1.1–3.1)**
*P*-value	**0.021**	**0.013**
Rating of how well the dental program prepared students for a successful career		
Yes, very well	**3.1 (1.4–6.8)**	**3.3 (1.5–7.0)**
*P*-value	**0.004**	**0.002**
Yes, somewhat	1.5 (0.7–3.2)	1.7 (0.8–3.6)
*P*-value	0.259	0.157
No, not enough	0.9 (0.4–1.9)	1.0 (0.5–2.1)
*P*-value	0.794	0.997
Perceptions of Private vs. Public Dental Schools: Education Quality & Career Prospects		
Worse	1.0 (0.7–1.5)	1.0 (0.7–1.5)
*P*-value	0.790	0.916
About the same	1.2 (0.9–1.7)	1.2 (0.9–1.7)
*P*-value	0.166	0.173
Better	**2.2 (1.5–3.1)**	**2.1 (1.5–3.0)**
*P*-value	**0.000**	**0.000**
Strengths of the dental education program		
Experienced faculty	**1.7 (1.3–2.2)**	**1.6 (1.2–2.1)**
*P*-value	**0.000**	**0.001**
Modern facilities &equipment	1.1 (0.8–1.5)	1.0 (0.7–1.3)
*P*-value	0.688	1.000
Practical/Clinical training	1.1 (0.9–1.5)	1.2 (0.9–1.5)
*P*-value	0.307	0.225
Research opportunities	1.3 (0.8–1.9)	1.2 (0.8–1.9)
*P*-value	0.274	0.366
Strong theoretical foundation	1.2 (0.9–1.5)	1.1 (0.9–1.5)
*P*-value	0.250	0.309
Important factors when considering a future career		
Job stability/security	**1.3 (1.1–1.7)**	**1.3 (1.0–1.7)**
*P*-value	**0.019**	**0.035**
Location	1.1 (0.8–1.4)	1.0 (0.8–1.3)
*P*-value	0.565	0.740
Opportunities for professional development	1.2 (0.9–1.5)	1.2 (0.9–1.6)
*P*-value	0.106	0.102
Reputation of institution/clinic	**1.4 (1.1–1.8)**	**1.3 (1.0–1.8)**
*P*-value	**0.009**	**0.027**
Salary &compensation	1.0 (0.8–1.2)	1.0 (0.8–1.2)
*P*-value	0.938	0.882
Work-life balance	1.1 (0.9–1.4)	1.2 (0.9–1.5)
*P*-value	0.342	0.203

Bold values are statistically significant.

OR, odds ratios; CI, confidence interval.

a Adjusted for demographics (Age, gender, marital status) and socioeconomic status (currently working (yes, no), study money source (own or parental money, scholarship, loan, or others).

Among the various strengths of the dental education program, only having experienced faculty showed a statistically significant positive association with preparedness (Adjusted OR = 1.6, 95% CI: 1.2–2.1, *P* = 0.001). Finally, regarding important factors when considering a future career, prioritizing “Job stability/security” was associated with 1.3 times higher odds of feeling well prepared (Adjusted OR = 1.3, 95% CI: 1.0–1.7, *P* = 0.035). Moreover, the “Reputation of institution/clinic” was also associated with better preparedness (Adjusted OR = 1.3, 95% CI: 1.0–1.8, *P* = 0.027). Other factors did not show a statistically significant relationship with preparedness in the adjusted analysis (Table 4).

## Discussion

The main findings of this study indicate that perceived dental students’ profession outlook and preparedness for practice were significantly associated with their perceptions of educational quality and their prioritized career-related values (such as job stability, salary, location, work-life balance and professional development). Positive evaluations of educational experiences and a strong sense of preparedness correlate with a more optimistic view of the profession. Whereas, concerns about job conditions or perceived advantages of private over public education may contribute to uncertainty or skepticism toward the dental profession.

This study offers crucial insight into the preparedness and career outlook of dental students in Iraq's private universities. Although most students reported feeling ready for professional practice, many expressed pessimism about the profession's future. These findings reveal a gap between educational outcomes and job market expectations, emphasizing the need for curriculum enhancement, stronger career guidance, and better workforce planning. The results provide essential evidence to support educational reform and policy development in Iraq's rapidly expanding private dental education sector.

An important finding from the study is the contrast between students’ confidence in their clinical preparedness and their pessimistic view of the dental profession's future. While many feel ready for practice, their outlook on career prospects remains largely negative. This suggests that, although current dental education effectively builds technical competence, it may fall short in preparing students for the economic and professional realities of the job market. The results highlight a need to enhance the curriculum by integrating career planning, market awareness, and entrepreneurial skills to better align students’ clinical readiness with realistic career expectations. Moreover, in contrast to our results, previous studies showed that dental students mostly exhibit optimism about their future profession outlook (12, 22).

These findings align with international efforts to strengthen dental education through competence-based frameworks such as the Association for Dental Education in Europe (ADEE) “Profile and Competences for the Graduating European Dentist,” the Commission on Dental Accreditation (CODA) standards, and the UK General Dental Council (GDC) learning outcomes. These frameworks emphasized not only clinical competence but also professional attributes such as communication, ethical judgment, teamwork, and employability. Adapting Iraqi dental curricula in line with these benchmarks could promote international equivalence and support students’ preparedness for both local and global dental practice environments (23–25).

Our results showed that work-related factors such as job stability, opportunities for professional development, salary, and work-life balance were related to perceived dental profession outlook. Prior research recognized the socioeconomic appeal of the dental profession (9). One study found that the main career goal among senior dental students is achieving financial stability. Work-life balance and high income/financial security were the main factors of long term goals (11). Long-term career plans embrace opportunities for professional development as well as personal issues such as work-life balance and financial outcome (7, 10).

The majority of dental students who participated in this study felt prepared for dental practice after graduation (either strongly or to a certain extent). This result is in line with previous studies that showed high self-perceived preparedness among undergraduate dental students (1, 2). However, one may argue that students usually overestimate their own readiness for professional practice (1, 4). Contrary to these studies, a more realistic view is that students feel less confident about practicing independently (6).

In our study, perceived dental students’ profession outlook and preparedness for practice are closely linked to their views on educational quality and their prioritized career-related values. Former research indicated that students often choose dentistry based on factors such as financial benefits, job security, and professional status, which significantly influence their expectations and preparedness for practice (8, 10). Moreover, high self-perceived preparedness for dental practice was associated with clinical experience and peer support (4). The curriculum as part of the dental education affects the self-perceived preparedness for independent dental practice. A study found that those trained under the traditional curriculum rated themselves as more prepared overall compared to those from the integrated curriculum (6).

The educational environment plays a central role in shaping students’ motivation, learning outcomes, and eventual professional identity. According to Biggs’ (1999) theory of constructive alignment, effective learning outcomes result when curriculum content, learning activities, and assessments are aligned with the intended professional competencies (26). In dental education, this includes not only acquiring clinical skills but also developing communication, ethical reasoning, decision-making, and lifelong learning capabilities (3). The extent to which educational experiences support these competencies may directly influence students’ readiness to enter practice or pursue specialization.

Bridging the gap between students’ confidence in their clinical abilities and their pessimistic perception of the dental profession requires a fundamental curricular shift toward employability and market awareness. Incorporating comprehensive career counseling is essential to give students a realistic understanding of the current Iraqi dental job market, addressing their priorities such as job stability, income, and work-life balance. Moreover, in light of market saturation and the rapid, often uncoordinated growth of private dental schools since 2003, the curriculum should include entrepreneurial training. This would prepare graduates not only to pursue existing employment opportunities but also to establish their own successful practices, broadening career options and enhancing financial resilience in a competitive landscape. Finally, strengthening communication skills is crucial, as these core professional competencies underpin effective patient management, team collaboration, and ethical decision-making, all of which contribute to professional success and a more optimistic long-term outlook (27–31).

Regarding the demographic and socioeconomic context of the participating dental students in this study, there was a dramatic change in dental education in Iraq that happened after 2003, particularly with the increasing licensing of private dental schools by the Ministry of Higher Education and Scientific Research. To date of this report, more than 60 private dental schools have been granted an official license in Iraq. Dental schools used to be exclusively public, and the number of accepted students in dental school was limited to the licensed public dental schools. The political change after 2003 brought change in the education direction towards private colleges and universities due to the high demand for medical specialties and an increased number of graduates from high schools and guaranteed governmental job opportunities after graduation (15, 16). It is worth mentioning that there is no coordination between The Iraqi Ministry of Higher Education and Scientific Research and the Ministry of Health concerning the number of accepted students in dental schools, which led to exacerbate the problem (15).

Furthermore, this study demonstrated that demographic and socioeconomic factors have an interplay effect between ‘Important factors when considering future career’ (location, opportunities for professional development, and reputation of the institution/clinic) and Self-perceived dental profession outlook. Previous studies highlighted the important effect of demographic and socioeconomic factors on the relationship between educational experiences and dental profession outlook (7, 8, 14, 20).

The high proportion of students perceiving the dental profession outlook as ‘Declining’ may be a direct reflection of concerns about market saturation due to the rapid, potentially uncoordinated, increase in private dental schools post-2003, as highlighted by previous research (15, 16). Several countries are suffering from the same phenomenon as Iraq, with unemployment among dental graduates (13, 32).

The High response rate from the participating institutions, providing robust data for the specific cohort studied. Although the concept of green dentistry promotes environmentally sustainable practices—such as reducing paper use, minimizing waste, and adopting digital technologies- the use of a paper-based questionnaire in this study is justified based on several methodological and practical considerations (33). First, by administering the questionnaire in paper format, inclusivity and the risk of excluding participants based on technological barriers were ensured. Secondly, paper-based questionnaires tend to yield higher response rates (which is the case in this study) in structured, face-to-face environments such as classrooms, and clinics at universities. This setting also allows the researcher to clarify any participant doubts, ensure that the questionnaire is completed on the spot, and minimize instances of incomplete responses. Furthermore, paper questionnaires reduce the likelihood of technical issues and distractions that are often associated with digital surveys. They also allow for full control over layout and visual formatting, reducing the chance of misinterpretation of questions due to screen variability. Nevertheless, the use of paper materials represents a limitation from a sustainability perspective. Future research in Iraqi dental education should consider adopting digital or hybrid survey methods that align with eco-friendly principles while maintaining data quality and accessibility, in keeping with the broader goals of green and sustainable dental practice (34). Additionally, using logistic regression models (multinomial and ordered) in the statistical analyses of the data gave the study robustness due to the control of the confounding factors (demographic & socioeconomic factors).

The possibility of self-report bias cannot be overlooked, as students may give desirable answers or underreport undesirable answers, which may lead to inaccurate data. For example, we noticed that a large proportion of students did not answer the question related to parents’ jobs. The language of the questionnaire was simple and easy to understand and free of technical terms or unclear words. We took into consideration the Middle Eastern culture when formulating the questions. Furthermore, questions were in two languages, English and Arabic, to avoid any misunderstanding. The study's high response rate can be attributed to two key factors: the significant interest shown by dental students in the personally relevant research topic, and the logistical support from teaching staff at the three universities who facilitated the distribution of the questionnaire prior to lectures and following clinical training. The participation in this study was anonymous and voluntary. These are effective methods for reducing self-report bias (especially social desirability bias). Assurances of anonymity and voluntary participation directly address respondent concerns about potential repercussions or judgment, encouraging more honest answers.

Large national cross-sectional studies that survey several public and private dental schools are needed to broaden our understanding of dental education and its connection to life after graduation among senior dental students. Future research should explore dental students’ plans for future practice (private, public, or other), their expectations regarding unemployment after graduation, including potential durations they might face, and the likelihood of them pursuing postgraduate studies, along with their specialty choices. It is also important to assess how likely they are to search for additional practical experience, such as internships and clinical training. Finally, studies on educational perspective differences between private and public dental schools are recommended.

## Conclusion

The results highlight the significance of enhancing educational quality, ensuring students feel career readiness, and addressing their career expectations to foster a more positive profession's outlook in Iraq.

## Data Availability

The raw data supporting the conclusions of this article will be made available by the authors, without undue reservation.
